# Supplementary material for: Which patients are more likely to experience compensatory hyperhidrosis after endoscopic thoracic sympathectomy: a meta-analysis and systematic review
Source: PeerJ. 2025 Mar 18;13:e19097. doi: 10.7717/peerj.19097 (PMC11927556; doi:10.7717/peerj.19097)
Supplement: Supplemental Information 1 [file peerj-13-19097-s001.docx]

**Audience Description Document**

**1. Research Background and Objectives**

This meta-analysis aims to evaluate which patients are more likely to experience compensatory hyperhidrosis (CH) and severe compensatory hyperhidrosis (SCH) following endoscopic thoracic sympathectomy (ETS) for hyperhidrosis. The findings of this study will provide scientific evidence to clinical practice, aiming to improve treatment outcomes and enhance the quality of life for patients with hyperhidrosis.

**2. Target Audience**

**2.1 Healthcare Professionals**

- **Thoracic Surgeons**: Particularly those who perform or are interested in performing ETS surgery will directly benefit from the results of this study to better assess and manage patients undergoing ETS.
- **General Practitioners and Family Physicians**: As they are often the first point of contact for patients seeking medical advice, understanding the risk factors for CH and SCH can help them provide more accurate counseling and referral recommendations.
- **Dermatologists**: Since hyperhidrosis is a common dermatological issue, dermatologists need to be aware of potential complications following ETS to offer comprehensive treatment options to their patients.

**2.2 Medical Researchers**

- **Epidemiology and Public Health Experts**: Interested in the epidemiological characteristics and influencing factors of hyperhidrosis, the data analysis provided by this study will aid them in further exploring the epidemiology of hyperhidrosis.
- **Basic Science Researchers**: The results may inspire further research into the pathophysiological mechanisms of hyperhidrosis, particularly regarding the autonomic nervous system and thermoregulatory responses.

**2.3 Patients and the Public**

- **Hyperhidrosis Patients**: They can directly benefit from this study by understanding the potential risks and outcomes following ETS, assisting them in making more informed treatment decisions.
- **Patient Support Groups and Advocacy Organizations**: They can use this information to educate patients and provide support, helping them better understand and manage their condition.

**3. Education and Training**

The results of this study can be incorporated into medical education and continuing professional development (CPD), helping healthcare professionals to update their knowledge base and raise awareness of complications following surgical treatment for hyperhidrosis.

**4. Policy Makers and Health System Planners**

Policy makers and health system planners can utilize this data to assess the demand for ETS surgery within public health systems and allocate resources accordingly, as well as to formulate guidelines and policies regarding the treatment of hyperhidrosis.
