# Supplementary figures and images for: Which patients are more likely to experience compensatory hyperhidrosis after endoscopic thoracic sympathectomy: a meta-analysis and systematic review
Source: PeerJ. 2025 Mar 18;13:e19097. doi: 10.7717/peerj.19097 (PMC11927556; doi:10.7717/peerj.19097)

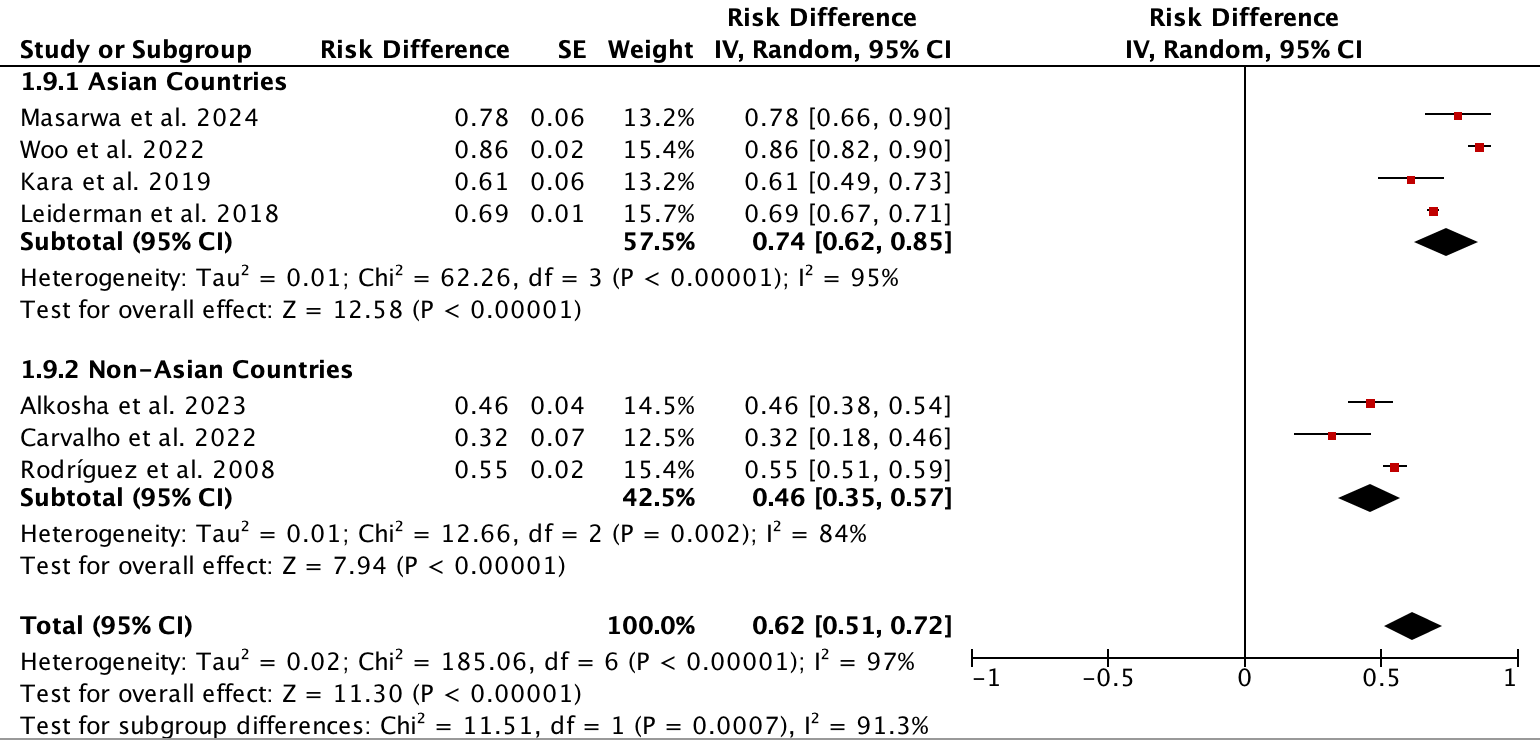

Supplement: Supplemental Information 3 [file peerj-13-19097-s003.tif]

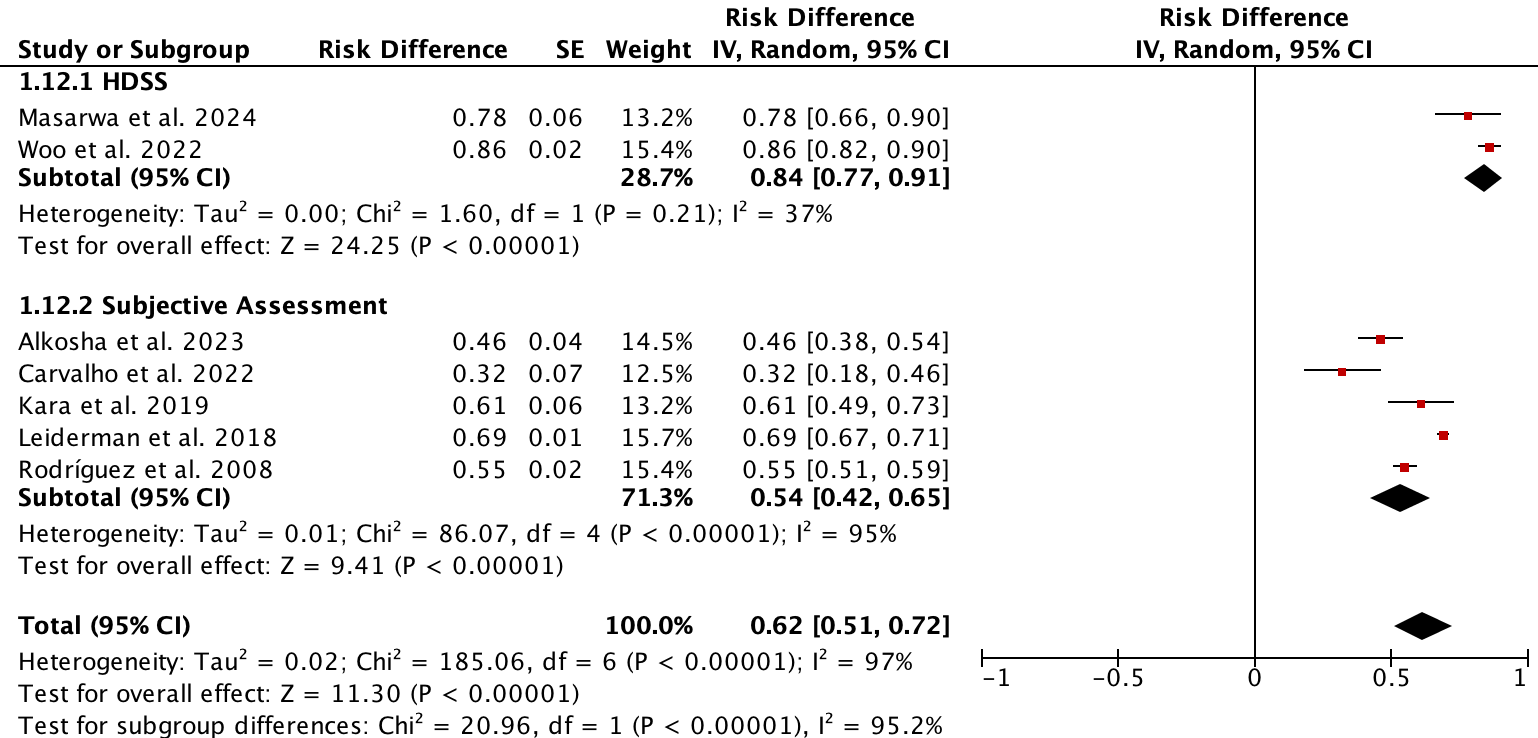

Supplement: Supplemental Information 4 [file peerj-13-19097-s004.tif]

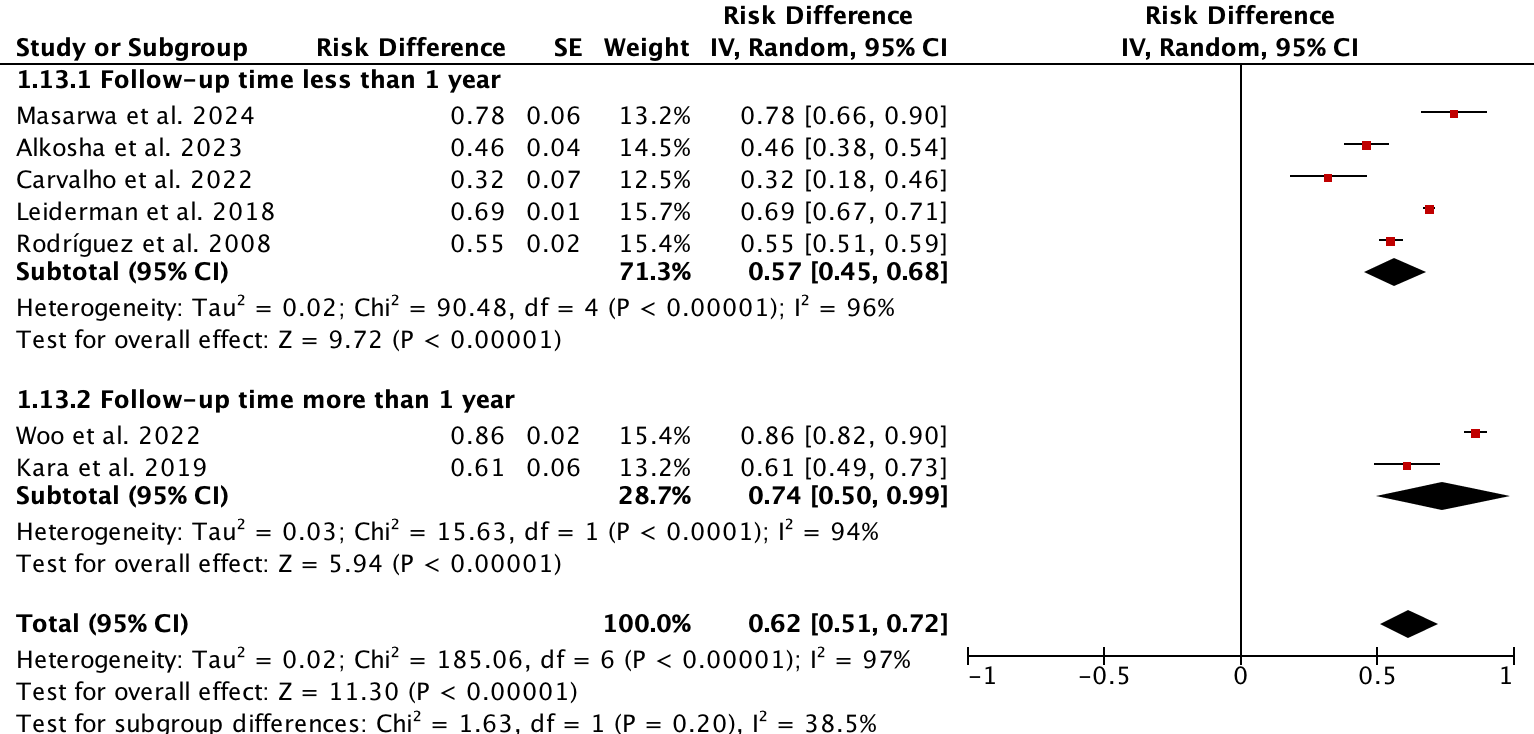

Supplement: Supplemental Information 5 [file peerj-13-19097-s005.tif]

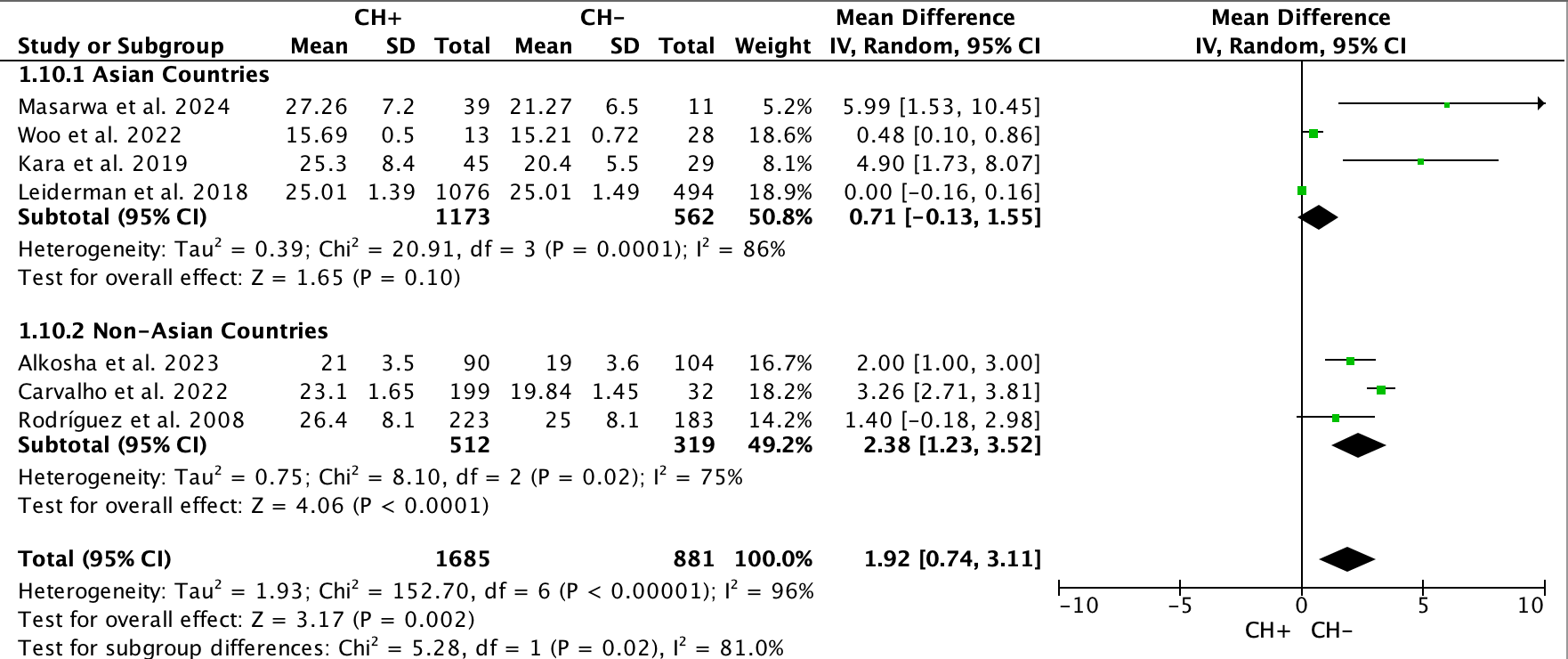

Supplement: Supplemental Information 6 [file peerj-13-19097-s006.tif]

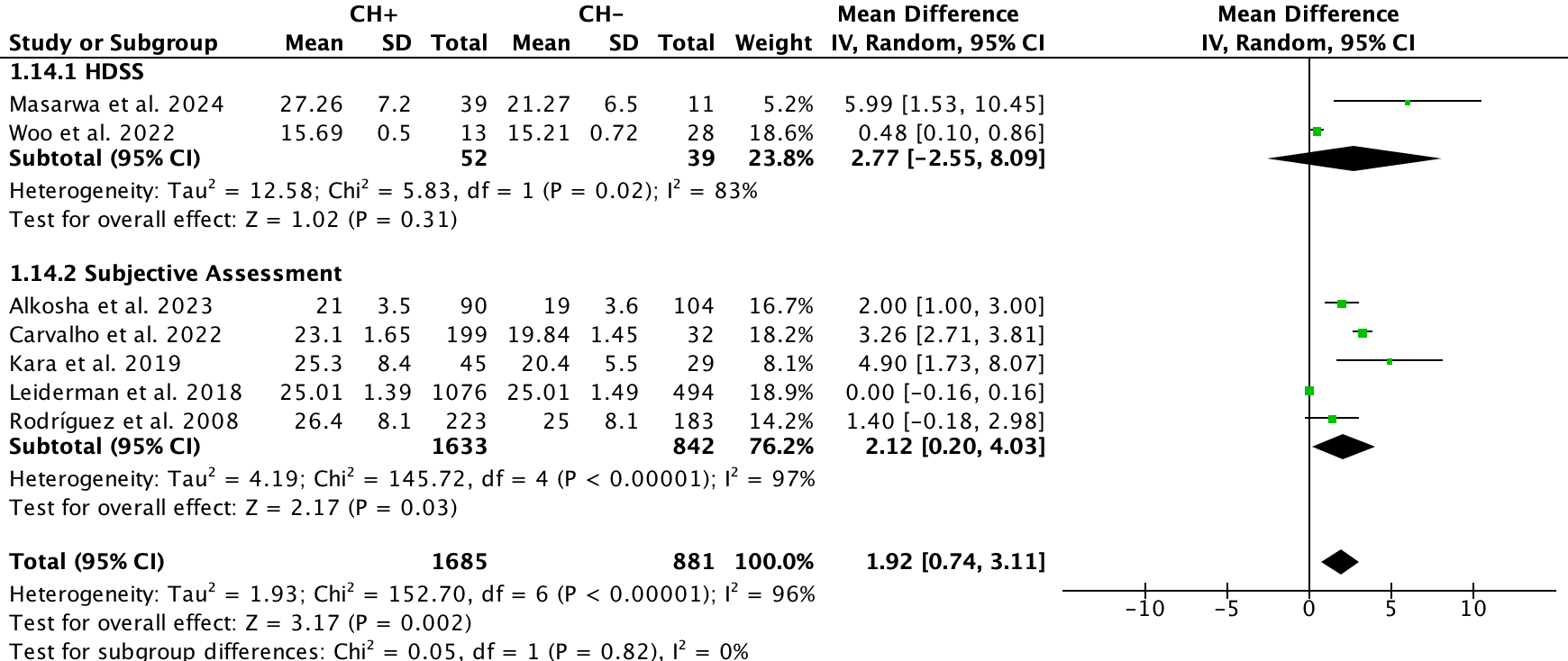

Supplement: Supplemental Information 7 [file peerj-13-19097-s007.tif]

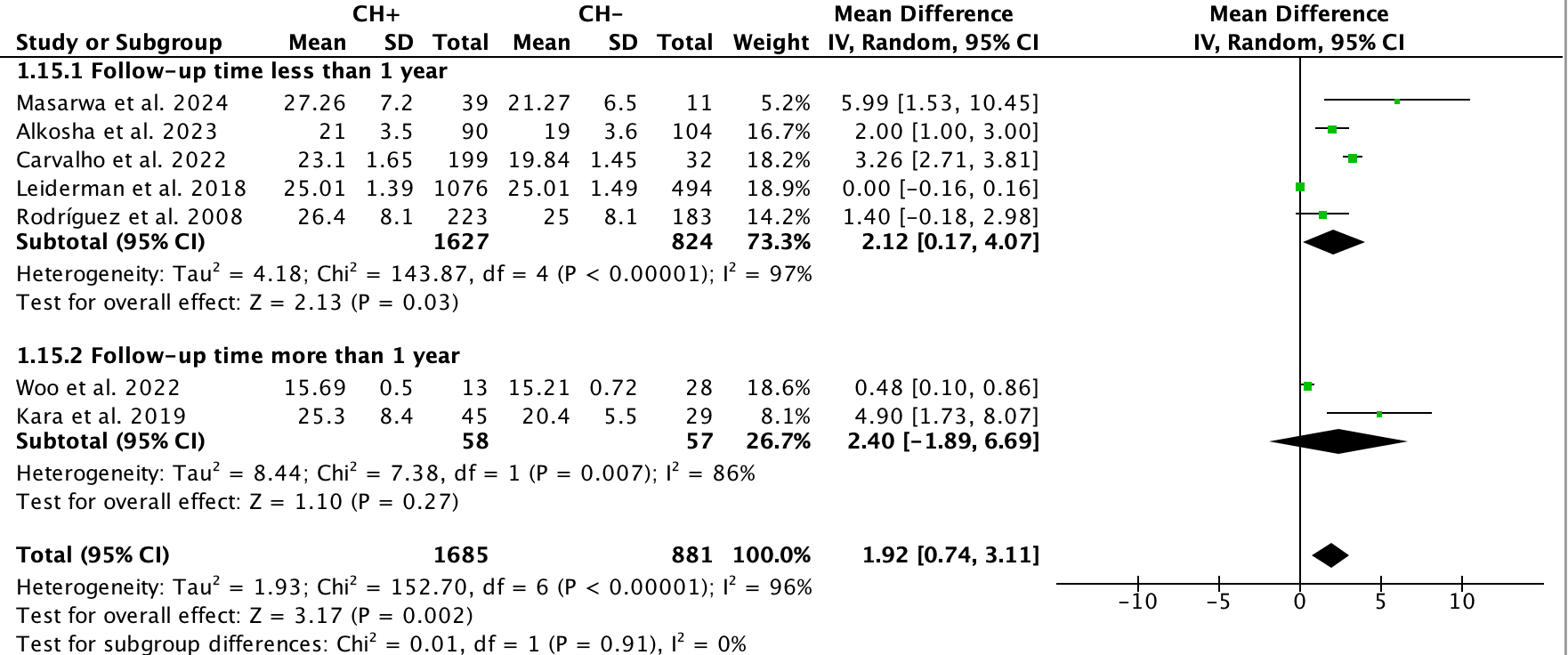

Supplement: Supplemental Information 8 [file peerj-13-19097-s008.tif]

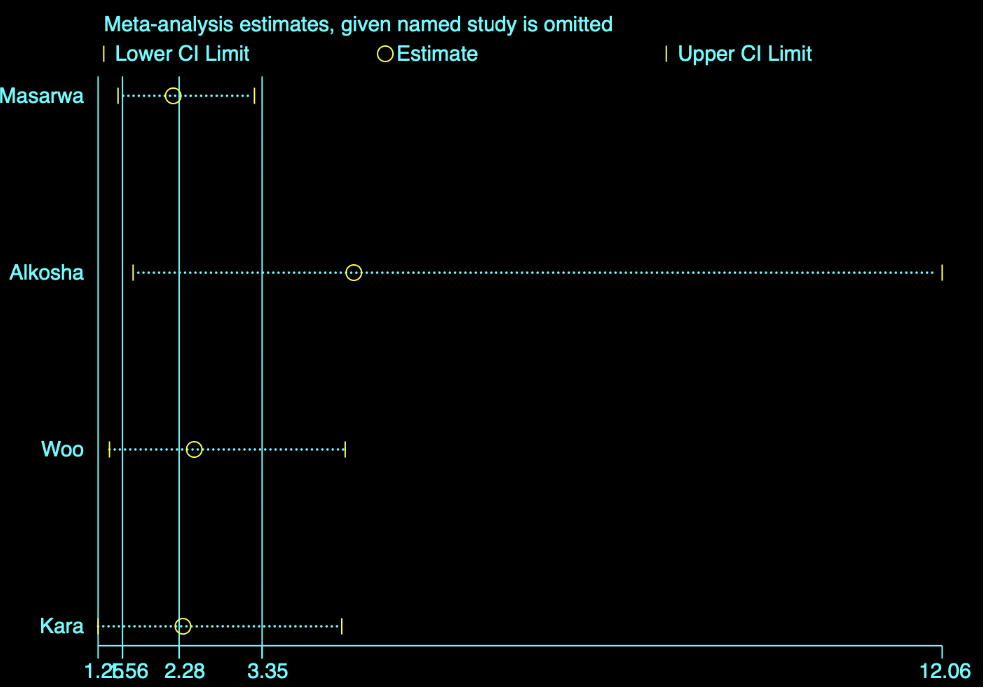

Supplement: Supplemental Information 9 [file peerj-13-19097-s009.tif]

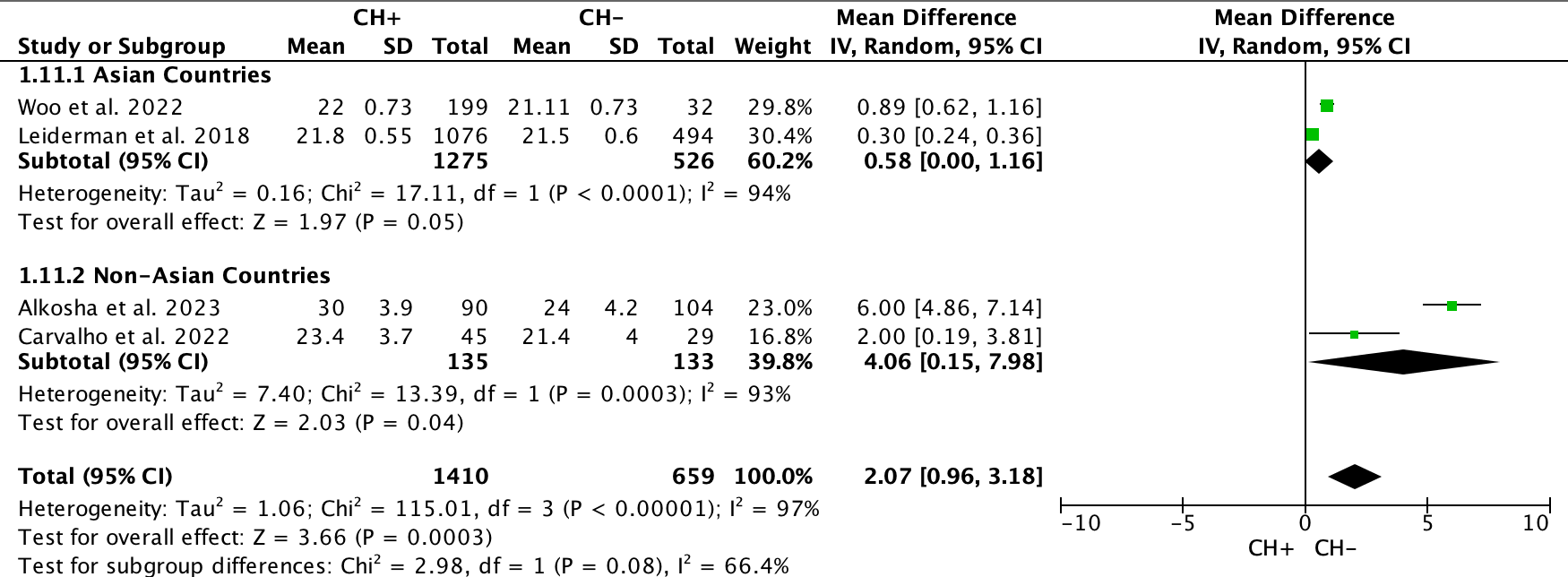

Supplement: Supplemental Information 10 [file peerj-13-19097-s010.tif]

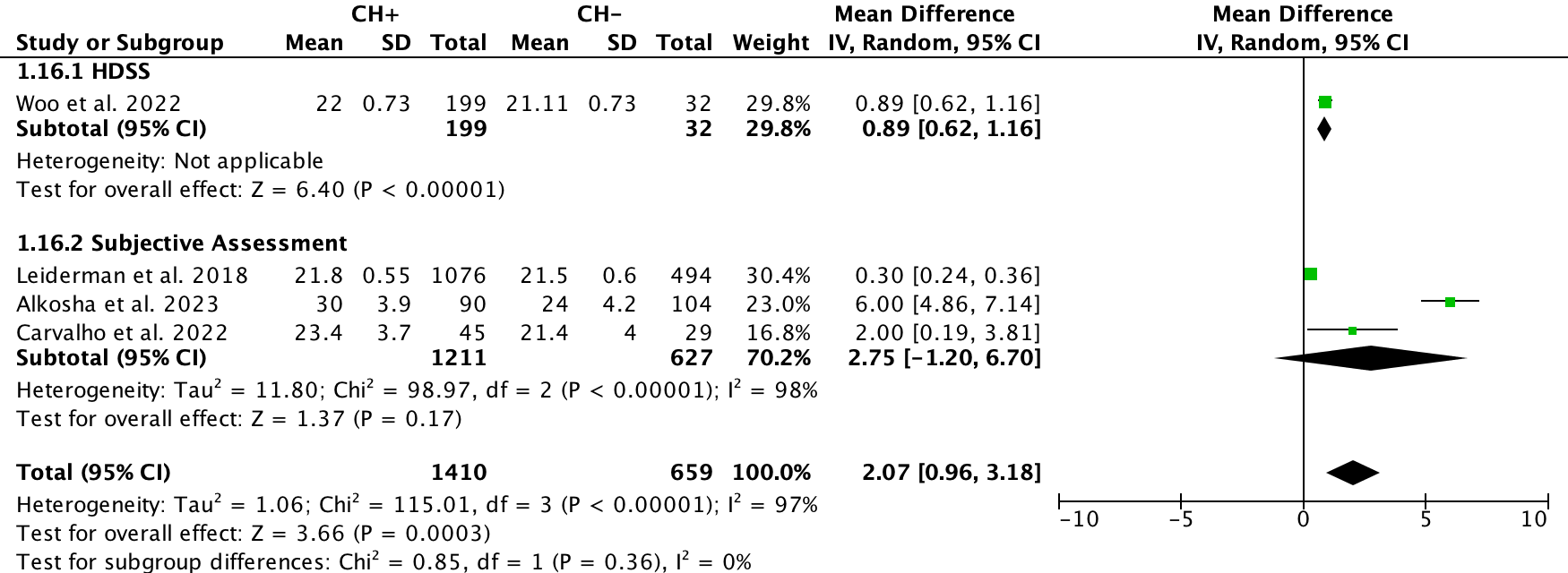

Supplement: Supplemental Information 11 [file peerj-13-19097-s011.tif]

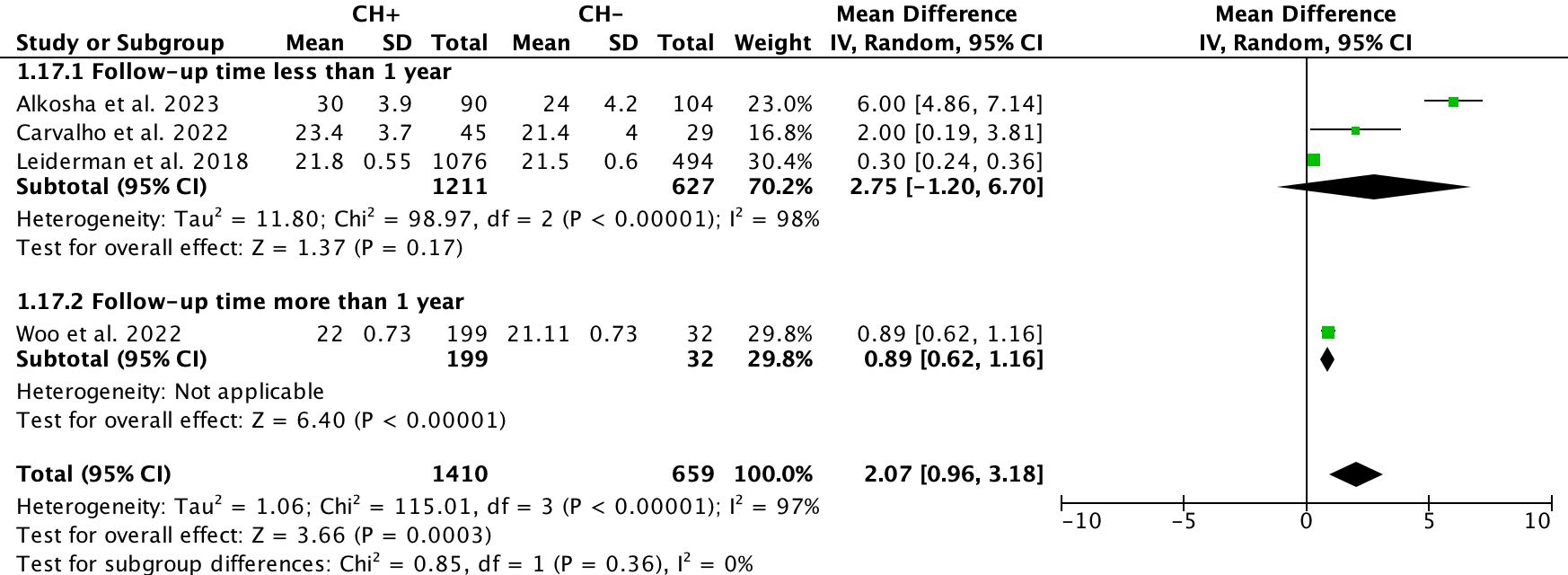

Supplement: Supplemental Information 12 [file peerj-13-19097-s012.tif]

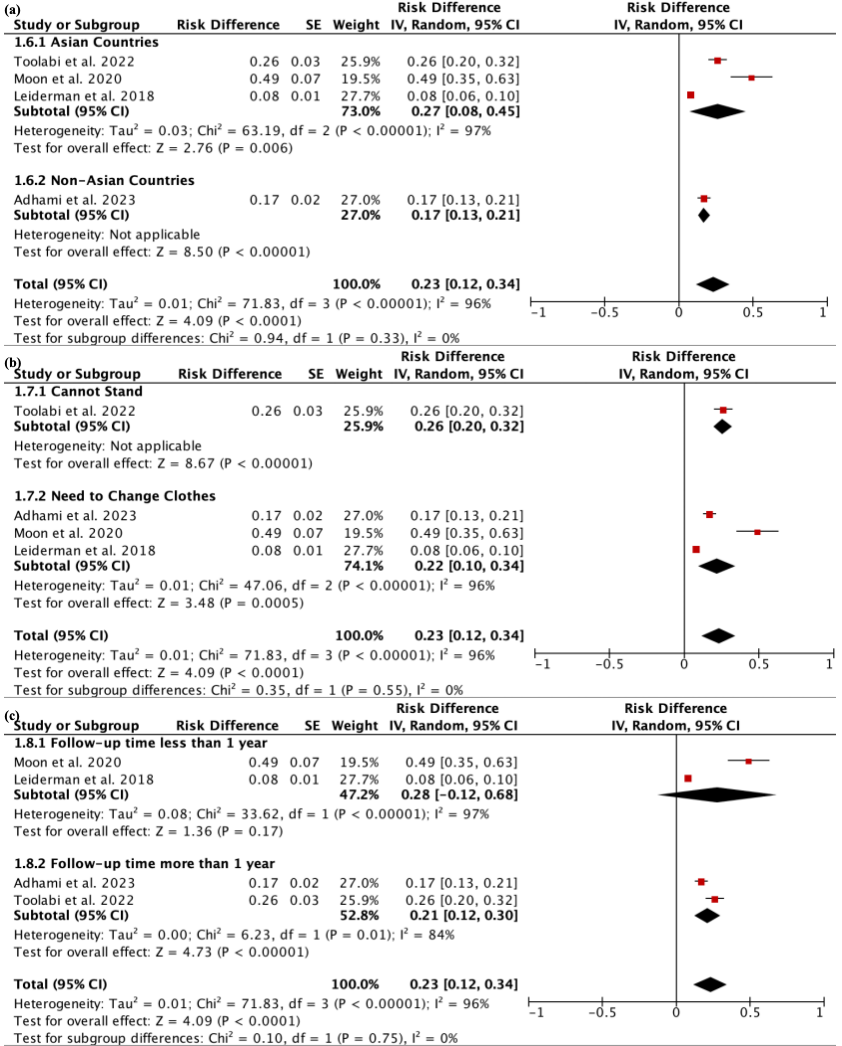

Supplement: Supplemental Information 13 [file peerj-13-19097-s013.tif]

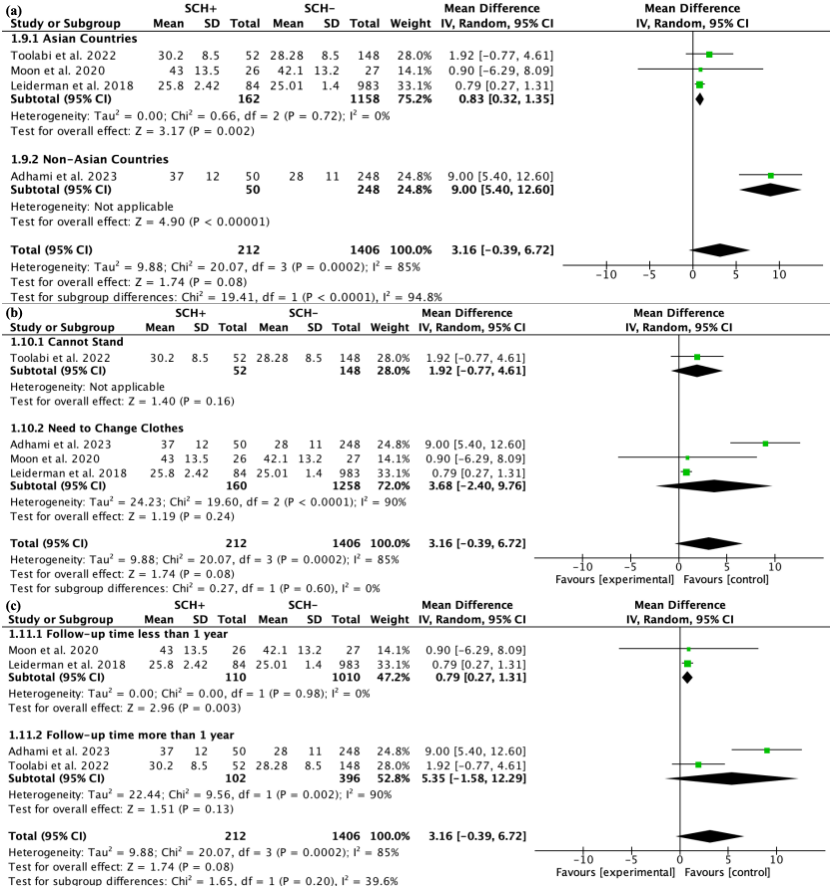

Supplement: Supplemental Information 14 [file peerj-13-19097-s014.tif]

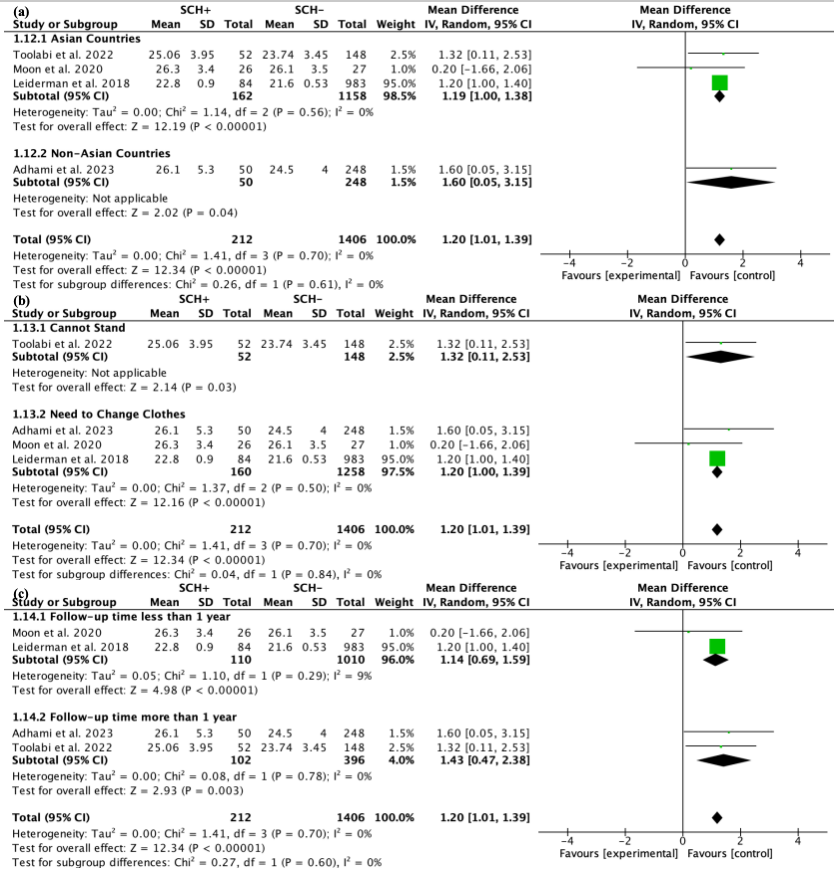

Supplement: Supplemental Information 15 [file peerj-13-19097-s015.tif]
